# Professional identity work in emerging healthcare professions: current perspectives, gaps, and future directions

**DOI:** 10.3389/frhs.2026.1778116

**Published:** 2026-02-24

**Authors:** Yiannis Kyratsis, Francesca Meda

**Affiliations:** 1 Erasmus School of Health Policy and Management, Erasmus University Rotterdam, Rotterdam, Netherlands; 2 CeRGAS, SDA Bocconi School of Management, Milan, Italy; 3 Sant’Anna School of Advanced Studies, Institute of Management, Pisa, Italy

**Keywords:** boundary work, emerging professions, healthcare workforce, identity work, legitimacy, person-centered care, professional identity, professionalization

## Abstract

Healthcare systems are witnessing the rapid emergence of new professional roles, including physician assistants, nurse practitioners, technical physicians, and healthcare data scientists. These occupational groups develop in contexts marked by role ambiguity, contested expertise claims, and fragmented organizational structures, making professional identity work—the processes through which individuals and groups construct, negotiate, and revise understandings of who they are as professionals—a central challenge shaping their development. This targeted conceptual review synthesizes current debates and identifies four strategic dilemmas that arise when emerging healthcare professions attempt to construct collective identity. These dilemmas concern whether to maintain flexible, generative identities or establish standardized, fixative definitions; whether collective professional identity emerges organically from practice or requires deliberate engineering in emerging professions where weak infrastructures leave unclear who has the authority to coordinate identity work; whether to position through differentiation claiming unique expertise or complementarity as essential partners; and whether to pursue jurisdictional control or relational accountability within interprofessional networks. Each dilemma is shaped by internal stratification along career paths, positional power, disciplinary traditions, and geographic locations, while professions simultaneously manage relationships with diverse external stakeholders including other professions, patients, policymakers, and the public. The review identifies critical research gaps concerning the recursive relationship between identity and practice, identity work proceeding without established occupational communities, the neglect of failed professionalization projects, and the underexplored material basis of professional expertise. Professional identity construction is fundamentally political. Understanding these dynamics is essential for workforce policy and professional development models that support person-centered care.

## 1 Introduction

Healthcare systems are witnessing the rapid emergence of new professional roles. Physician assistants, nurse practitioners, clinical genomics specialists, technical physicians, and healthcare data scientists now populate a landscape once dominated by established professions with centuries of institutional scaffolding. These new roles arise from technological innovation, expanding knowledge bases, evolving care models, persistent workforce shortages, and shifting patient expectations. They develop in contexts marked by role ambiguity, contested expertise claims, and fragmented organizational structures. These roles are central to emerging models of person-centred care that require professionals capable of working flexibly across traditional boundaries.

A core challenge shaping the development of new occupations is professional identity work: the processes through which individuals and occupational groups construct, negotiate, and revise understandings of “who we are” and “who we are becoming” as professionals. For established professions, identity work proceeds against a backdrop of shared institutional resources—professional associations, socialization, jurisdictional mandates, clear prototypes of what it means to be a doctor, nurse, or lawyer. Emerging professions lack these scaffolds. Their practitioners must collectively define shared values while simultaneously presenting themselves coherently to skeptical external audiences.

This review synthesizes current debates and identifies four strategic dilemmas that arise when emerging healthcare professions attempt to construct collective identity. These dilemmas are not merely theoretical abstractions. They emerge from the practical necessity of navigating, simultaneously, diverse practitioners positioned along structural dimensions of career paths, power asymmetries, disciplinary traditions, and geographic locations, together with multiple external stakeholders—other professions, patients, policymakers, the public—each evaluating them according to their own priorities and concerns.

The four dilemmas concern whether to maintain flexible, generative identities or stick to standardized, fixative definitions; whether identity should be coordinated organically or be engineered through (still developing) formal structures; whether to position through differentiation claiming unique expertise or complementarity as essential partners; and whether to pursue jurisdictional control or relational accountability within interprofessional networks. Each represents genuine strategic options with real consequences, revealing identity formation as both cognitive and political project requiring navigation of internal conflict while managing external legitimacy. The review also identifies critical gaps in existing literature and outlines future research directions for workforce policy and professional development.

## 2 Review approach and scope

We adopted a targeted review privileging conceptual integration over exhaustive coverage, consistent with the Mini Review format. Using Web of Science, Scopus, and Google Scholar we searched combinations of “professional identity,” “occupational identity,” “identity work,” “boundary work,” “role transition,” “emerging professions,” “healthcare professionals,” and “technology,” supplemented by citation tracing. Inclusion was restricted to peer-reviewed English-language articles and books published since 2000 theorizing or empirically examining professional identity in healthcare or closely related expert professions. We excluded purely clinical, technical, or educational studies without theoretical engagement with identity processes. We emphasized professions such as physician assistants, technical physicians, and healthcare data scientists as illustrative cases because they offer theoretically rich cases where new roles, technologies, and institutional logics intersect, making identity construction processes particularly visible.

## 3 The centrality of professional identity work in emerging professions

Healthcare is experiencing rapid proliferation of new professional roles—physician assistants (1), nurse practitioners (2), technical physicians (3), healthcare data scientists (4). This proliferation reflects technological innovation (5, 6), knowledge disruptions (7), evolving care models (8), workforce shortages (9), and changing patient expectations (10, 11). The outside world increasingly penetrates professional domains, through volatile labor markets (12, 13), less stable careers (14), fragmented education pathways (15), expanding specialization (16), more knowledgeable patients (17), transnational service provision (18, 19), and greater exposure of professional failures (20). Professional work now faces heightened control (19) and permeable boundaries (12).

In response, scholars have examined how professionals adapt their identities in changing contexts (21). Professional identity is understood as “an individual's self-definition as a member of a profession and is associated with the enactment of a professional role” (22), serving as a framework through which individuals assign meaning to themselves (23), fuel their self-esteem (24), and claim purpose (25). For professionals, whose identities are closely tied to their work practices (22, 23), understanding the interplay of professional work and professional identity is particularly important. Rather than viewing identity as fixed, scholarship [e.g., (26–28)] conceives it as ongoing construction requiring active work, especially during contextual change or role transitions (29).

Identity work describes activities to create and sustain identities congruent with self-concept. Scholars have investigated how professionals adopt established role identities when transitioning careers (23), reconcile multiple logics (30, 31), respond to identity threats (32), and adapt to societal (33), institutional (21), and technological change (34, 35). Yet this literature has concentrated on long-established professions such as law (62), medicine (21, 36, 37), academia (38), banking (39), and management consulting (40). Far less attention addresses how professionals construct identity when their profession is still in early stages, precisely when identity work is more demanding.

Emerging professionals must collectively define shared values and norms (41), construct occupational mandates distinguishing them from others and establish the grounds for legitimating jurisdiction (42). Yet they lack the identity resources established professions possess, i.e., professional associations, socialization rituals, jurisdictional mandates, routines, identity prototypes, and distinctive professional logic (43). Critically, practitioners within these occupations are stratified along four structural dimensions that create divergent interests in how collective identity should be constructed.

The first dimension concerns *career paths and institutional locations.* Practitioners pursue academic, clinical, industry, or policy careers in contexts operating under different institutional logics, incentives, and constraints. A healthcare data scientist building algorithms in a technology startup faces different pressures than one embedded in a hospital quality improvement unit or one pursuing an academic career in health informatics. These structural positions shape professional interests independently of individual preference.

The second dimension involves *differential positional power and institutional investment*. Early leaders, especially those occupying formal positions within emerging professional structures, have invested institutional capital in foundational identity choices and face reputational risk if those choices are reopened. Newcomers with less institutional investment can afford greater risk and may resist premature fixation. Risk-averse actors, often profession leaders, may prefer premature standardization to secure immediate legitimacy and clear credentials; risk-tolerant practitioners prefer flexibility for potentially greater future authority.

The third dimension reflects *disciplinary and knowledge traditions.* Practitioners trained in medicine, nursing, computer science, bioinformatics, or public health bring different epistemologies about what constitutes legitimate expertise and professional authority. These disciplinary commitments persist and shape disagreements about professional identity content and scope.

The fourth dimension encompasses *geographic and jurisdictional locations.* Practitioners distributed across different regulatory, policy, and healthcare system contexts face divergent pressures creating conflicting interests in standardization pace, credentialing models, and positioning.

These dimensions create persistent internal stratification. Without shared institutional scaffolding, emerging professionals must achieve internal consensus while presenting coherently to external stakeholders, despite conflicting preferences for strategic options due to structural reasons (different career paths, power asymmetries, disciplinary traditions, geography). The political challenge is substantial, bridging internal stratification for achieving consensus on identity positioning while managing diverse external stakeholder expectations embedded in sociotechnical.

## 4 Four strategic dilemmas: collective professional identity work

We identify four strategic dilemmas that emerging professions navigate during collective identity construction. These are not abstract theoretical questions, but genuine options shaped by internal stratification and external stakeholder management.

### 4.1 Generative vs. fixative identity trajectories

The first dilemma concerns whether professions should maintain flexible, generative identities that evolve with practice and accommodate diverse career pathways, keeping future options open, or establish standardized, fixative identities with clear credentials that restrict the option set but provide coherence to external stakeholders. Generative approaches preserve innovation and adaptability, allowing professions to respond to unanticipated developments in practice and knowledge. Nonetheless, they risk appearing incoherent to policymakers and established professions seeking clarity about what the profession is and does. Fixative approaches signal maturity and institutional legitimacy through standardization, but early decisions to standardize prematurely constrain what becomes organizationally possible later, potentially foreclosing alternative professional configurations and narrowing the range of practitioner profiles the profession can accommodate.

### 4.2 Emergent vs. engineered identity formation processes

The second dilemma addresses coordination capacity during formation. In established professions, professional associations, credentialing agencies, and academic programs provide infrastructure for orchestrating collective identity. Emerging professions lack this scaffolding. Associations may be nascent or fragmented, credentials unclear or borrowed from parent disciplines, academic programs dependent on other departments for legitimacy, and practitioner forums poorly resourced. The dilemma is not whether identity is engineered—some orchestration is always occurring—but who has authority (and credibility) to coordinate identity work when no established infrastructure exists, and how professions build coordination capacity while simultaneously requiring coordination to do so. Early identity decisions made by weakly mandated actors may lack legitimacy, while waiting for stronger infrastructure risks ceding ground to external actors or allowing fragmentation to become entrenched.

### 4.3 Differentiation vs. complementarity positioning

The third dilemma concerns positioning strategy, how they frame the profession publicly. Emerging professions must decide which narrative to prioritize when presenting themselves to multiple audiences. Should they emphasize unique expertise and autonomous professional status? Or should they lead with a complementarity narrative, positioning themselves as essential partners filling gaps in existing healthcare delivery systems? A differentiation-led narrative creates the necessity for new organizational positions, job roles, and departmental structures. It forces healthcare organizations to create actual spaces and positions. However, this path carries significant structural consequences. It may provoke defensive reactions from established professions viewing the emerging profession as competitive threat, creating conflict and resistance that can substantially delay or prevent acceptance. A complementarity-led narrative positions the profession as filling gaps within existing systems without requiring new structures. This avoids direct competition and may gain political acceptance more readily. However, this path carries a more fundamental structural problem. If the emerging profession's work is genuinely novel there may be no actual organizational slots available even if the profession is in-theory accepted. Practitioners face a paradox of achieving political acceptance while having nowhere to work, because complementarity assumes the work can be accommodated within existing structures when it fundamentally may not.

### 4.4 Jurisdictional vs. relational professional control

The fourth dilemma concerns whether identity work should center on jurisdictional control (controlling specific tasks, knowledge domains, protecting boundaries against encroachment), or relational accountability (performing reliably within interprofessional networks, building relationships of mutual respect and interdependence). Jurisdictional approaches provide clear professional boundaries and protect against boundary erosion by other professions. They clarify what the profession claims authority over. Relational approaches align with contemporary healthcare emphases on collaboration and person-centered care and build broader support among external stakeholders. However, they require practitioners to subordinate individual professional interests to collective goals and may leave the profession vulnerable to boundary erosion over time.

### 4.5 Navigating dilemmas amid internal stratification

Research reports cases where strategic choices have proven productive. Studies about nurse practitioners and physician assistants demonstrate how deliberately structured professional identities supported by standardized education and credentialing have facilitated legitimacy and integration into care delivery systems (44–46). Research on hybrid and extended roles suggests negotiated professional identities can serve as enabling structures supporting interprofessional collaboration and accountability (47–49). Under certain conditions, identity consolidation stabilizes novel work arrangements and provides platforms for professional development (23, 50). These cases suggest that strategic clarity and deliberate standardization, when appropriately timed, can enhance rather than constrain professional development, securing legitimacy and improving care quality. This raises a critical question remaining underexplored: when and how does identity stabilization enable rather than constrain emerging professions (46, 51, 52)?

These dilemmas involve genuine tradeoffs substantially amplified by internal stratification. Practitioners in academic careers may prefer standardized credentials and differentiation supporting academic recognition and disciplinary legitimacy. Clinicians embedded in hospitals may favor flexible, emergent identity allowing adaptation to local patient needs and relational accountability within care teams. Industry practitioners may prioritize rapid standardization for market scalability. Policy-oriented practitioners may emphasize complementarity to secure regulatory recognition. Those with greater positional power and institutional investment may prefer stability; newcomers may prefer flexibility. Different disciplinary traditions create epistemic conflicts about legitimate expertise. Geographic distribution can create competing interests in standardization pace.

**Figure 1:**
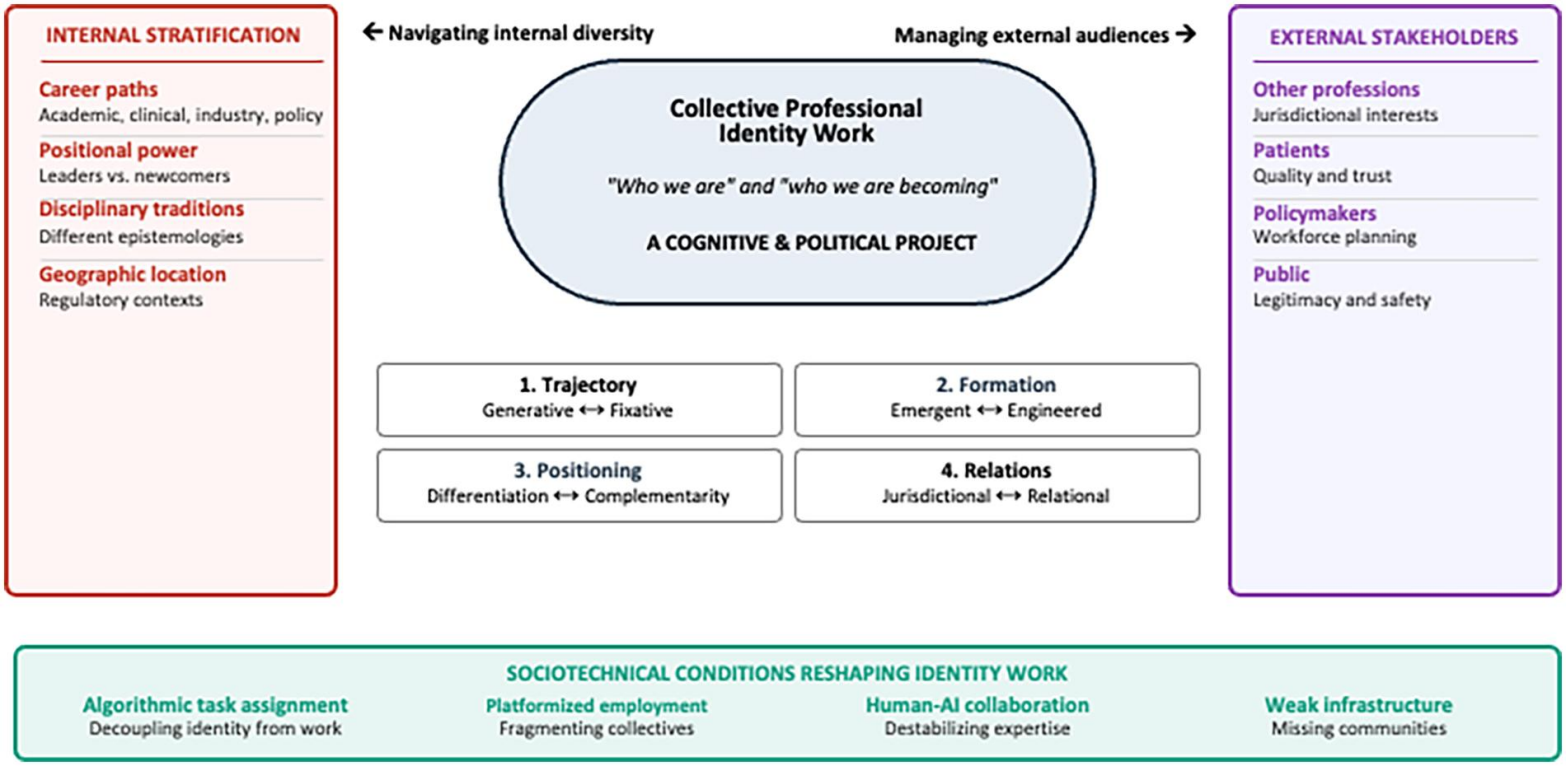
Collective professional identity work in emerging healthcare professions. This figure presents collective professional identity work as a cognitive and political project requiring emerging healthcare professions to navigate internal stratification while managing external stakeholder expectations. The core challenge of constructing shared understandings of “who we are” and “who we are becoming” as professionals is shaped by four strategic dilemmas: generative vs. fixative trajectories, emergent vs. engineered formation, differentiation vs. complementarity positioning, and jurisdictional vs. relational professional control. Internal stratification (left) identifies structural dimensions creating divergent practitioner interests; external stakeholders (right) represent audiences with competing evaluation criteria. Sociotechnical conditions (bottom) identify material forces reshaping both the dilemmas and the profession's capacity to navigate them collectively.

The cognitive and political project of collective identity work involves navigating these structural conflicts while simultaneously presenting coherent positioning to external stakeholders. Successful navigation may depend on mechanisms emerging informally within professional communities—trusted leaders bridging practitioner groups and creative brokering acknowledging internal diversity rather than erasing it. Figure 1 synthesizes these dynamics, situating collective professional identity work at the intersection of internal stratification and external stakeholder management. The figure illustrates how practitioners positioned differently along structural dimensions hold divergent preferences for navigating each dilemma, while external stakeholders evaluate the emerging profession according to their own priorities. Sociotechnical conditions further complicate this landscape, altering both the dilemmas and the internal capacity for collective action.

## 5 Critical gaps in existing research

Four research gaps limit understanding of collective identity work in emerging professions. First, the recursive relationship between identity and practice remains poorly understood. Identity-centric approaches tend to assume practice as backdrop, with identity responding to changes in work (53, 54), while practice-centric approaches posit that meaning and identity necessarily emerge from practices and through practices (55). Whether identity and practice constitute one another recursively rather than operate sequentially remains unclear. This recursive relationship complicates strategic choices because decisions to standardize identity may constrain what becomes organizationally possible in practice, which then reshapes identity demands in unexpected ways.

Second, practitioners in emerging professions lack established occupational communities typically supplying raw materials for identity construction, i.e., shared values, legitimating ideologies, routines, collectively recognized tasks (56). Practitioners often remain occupationally isolated, given little prepackaged identity content (57). How does then identity work proceed without such anchoring structures? The absence of occupational communities makes consensus-building among structurally diverse practitioners harder, intensifying internal political conflict precisely when coordination is most essential.

Third, scholars remain divided on the temporality, whether identity unfolds toward equilibrium (63), or persists as ongoing process without predetermined endpoints (58, 59). Moreover, research exhibits a critical blind spot in examining cases that successfully professionalize while largely ignores cases where collective identity projects fail or collapse. Understanding failure could illuminate conditions enabling success.

Fourth, materiality receives scant attention despite its centrality to emerging healthcare professions. These professions depend on rapidly evolving technologies that can destabilize the very basis of professional expertise. When the material basis of professional knowledge becomes obsolete within years rather than decades, established boundary work strategies may prove insufficient. Recent research suggests professionals respond to material disruption through boundary work aimed at protecting jurisdiction while extending scope (60, 61). However, how these processes unfold in emerging occupations where identity itself remains unsettled and contested remains unclear (5).

## 6 Implications and future directions

Understanding professional identity work in emerging healthcare professions requires recognizing it as fundamentally cognitive and political. Emerging professions face distinctive challenges as material and employment conditions shift. Algorithmic technologies assigning tasks based on efficiency metrics rather than professional jurisdiction further complicate the jurisdictional-relational. If algorithms determine what work gets done, jurisdictional claims become difficult to sustain. Platformization and gig-like employment arrangements can fragment collectives across geographic and institutional boundaries, making consensus harder to achieve. Such material and employment conditions create a challenging situation; coordination and collective agreement are hardest to achieve precisely when the consequences of early identity choices are greatest.

For workforce policy and healthcare planning, these dynamics carry significant implications. Person-centered care requires professionals who work flexibly across professional boundaries, adapt to evolving patient needs, and integrate diverse knowledge sources. These capabilities may characterize emerging professions with fluid, adaptive identities more readily than established professions constrained by rigid jurisdictional boundaries. For person-centred care, this suggests emerging professions oriented toward relational accountability rather than jurisdictional defense may be particularly well-suited to responsive, individualized practice. Navigating this landscape presents genuine tensions that cannot be resolved through simple choices. Adaptability and flexibility must coexist with sufficient grounding in professional standards and ethical commitments to ensure quality and safety. All four dilemmas present such simultaneous demands requiring careful navigation rather than decisive resolution.

Future research should address three interconnected directions. First, how do emerging professions achieve sufficient internal consensus on strategic dilemmas despite structured diversity? What role do trusted leaders and informal networks play in bridging different practitioner groups? Second, how do algorithmic, platform-based, and human-AI work arrangements reshape both the strategic dilemmas emerging professions face and the structural dimensions of internal stratification itself? For fragmenting employment arrangements what new coordination forms become necessary? Third, how can emerging professions navigate tensions between needing strategic clarity for external legitimacy and resource acquisition while maintaining sufficient flexibility to accommodate internal diversity and respond to evolving contextual needs?

Addressing these questions research should attend to both documented cases where professions have managed dilemmas productively and underexplored cases where collective identity projects have failed or collapsed, as understanding failure illuminates conditions enabling success. This understanding is particularly urgent as contemporary healthcare systems pursue increasingly complex models of care requiring new forms of professional collaboration and identity.
